# Aberrant sialic acid metabolism promotes metabolic reprogramming and metastasis in breast cancer

**DOI:** 10.3389/fonc.2025.1698087

**Published:** 2025-11-17

**Authors:** Longyu Yang, Zhenhua Li, Meijin Wang, Xing Xiong, Shilin Zhang, Zhi Man, Chunyan Dong, Li Fu

**Affiliations:** 1 Shanghai Clinical Research and Trial Center, ShanghaiTech University, Shanghai, China; 2 School of Life Science and Technology, ShanghaiTech University, Shanghai, China; 3 Department of Oncology, WeiFang People’s Hospital, Shandong Second Medical University, Weifang, Shandong, China; 4 Department of Oncology, East Hospital Affiliated to Tongji University, Tongji University School of Medicine, Shanghai, China; 5 Department of Breast Cancer Pathology and Research Laboratory, Tianjin Medical University Cancer Institute and Hospital, Tianjin, China

**Keywords:** breast cancer, sialylation, metabolic reprogramming, metastasis, Neu5Ac

## Abstract

**Background:**

Dysregulated tumour metabolism is increasingly recognised as a central driver of malignant phenotypes. Against this background, the aberrant metabolism of N-acetylneuraminic acid (Neu5Ac), a core constituent of the sialic acid family, and its impact on breast cancer progression is now receiving significant research attention.

**Methods:**

The purpose of this study is to employ metabolomic approaches to analyze and interpret differences in metabolite profiles connected to breast cancer. Following this, a comprehensive multi-omics analysis will be employed to reveal the differences at transcriptional and metabolic levels in cells after the addition of external sialic acid. Finally, modification proteomics will be applied to recognize and characterize proteins that have different sialylation patterns.

**Results:**

Cells treated with sialic acid showed improved motility, underwent metabolic reprogramming, and experienced a significant rise in the sialylation levels of key proteins.

**Conclusion:**

This study collectively elucidates the role of Neu5Ac metabolism in promoting breast cancer invasion and metastasis through the remodeling of lipid metabolic pathways and alterations in protein sialylation. The findings present novel evidence supporting the targeting of sialic acid metabolism and its modifications as potential therapeutic strategies for inhibiting tumor progression.

## 1 Introduction

Post-translational modifications (PTMs) constitute a fundamental molecular mechanism for the regulation of protein function. These modifications take place subsequent to protein synthesis, introducing chemical changes that alter a protein’s three-dimensional structure and biological activity (1, 2). PTMs are pervasive within cells and encompass major classes such as phosphorylation, acetylation, ubiquitination, and glycosylation (3, 4). Glycosylation, a particularly complex form of PTM (5), is distinguished by the absence of direct genetic encoding for its glycan component, unlike the primary structure of proteins. For this reason, elucidating the precise correlation between the structural arrangement of glycans and glycoconjugates and their functional roles has emerged as a key research imperative in the post-genomic era.

UDP-GlcNAc plays several crucial biological roles within the cell: it acts as the immediate donor substrate for O-GlcNAcylation (6) and is also an essential precursor for sialic acid biosynthesis (7). Research has demonstrated that UDP-GlcNAc is involved, either directly or indirectly, in the biosynthesis of various glycan moieties, including fucose, mannose, and notably, sialic acid. Notably, levels of sialic acid have been observed to increase significantly in conjunction with the upregulation of UDP-GlcNAc (8). Aberrant sialylation has been strongly implicated in the pathogenesis and progression of numerous diseases, including malignancies (9), neurodegenerative disorders (10), and immune system dysfunctions (11). Sialyltransferases facilitate the sialylation process by covalently linking sialic acid to the terminal regions of glycoproteins and glycolipids, producing sialoglycans (12). Sialic acid can subsequently be removed by neuraminidases in a process known as “desialylation”. Sialylglycans are predominantly situated on the outermost layer of the cell surface, where they play crucial roles in biological processes, including neoplastic transformation, proliferation, metastasis, and immune evasion (13).

As a charged glycan, excessive accumulation of sialic acid on the cell membrane disrupts the stability of cell-cell adhesion via electrostatic repulsion (14, 15). Research suggests that sialylation modulates the adhesive and migratory capacities of breast cancer cells by altering the glycosylation patterns of cell surface proteins. Specifically, α-2,6-sialylation, facilitated by ST6GAL1, enhances the migration of breast cancer cells across pulmonary endothelial monolayers, thereby elevating the risk of lung metastasis (16). Additionally, increased expression of ST8SIA4 is associated with aggressive behavior in breast cancer cells, as it regulates cellular proliferation and invasive potential by modulating sialylation levels on cell surface glycoproteins (17). Beyond affecting the migration of cells, sialylation influences cancer progression through regulatory effects on signaling pathways. Studies have shown that sialylation can augment the activity of cell adhesion molecules, such as integrins, thereby promoting tumor cell invasion and metastasis (18, 19). Furthermore, through activating integrins to pull ECM ligands, it promotes the mesenchymal-like phenotype of cells and may also exert an effect on the cell cycle (14). Additionally, sialylation contributes to tumour immune evasion mechanisms. It facilitates this by masking neoantigens and epitopes, thereby reducing recognition and attack by the immune system (20–22). It is also possible to achieve this through truncation of the mucin-type O-linked glycans (23). Alterations in sialic acid metabolism significantly affect intercellular adhesion and signaling, and they are also pivotal in tumor growth and dissemination. In the realm of breast cancer, metabolomics investigations have demonstrated a significant association between heightened sialic acid metabolism and highly metastatic tumors (24). Furthermore, the use of unnatural sialic acid precursors has resulted in a significant decrease in both natural and polysialic acid expression within breast cancer cells. This observation suggests that modulating sialic acid metabolism can influence tumor cell biology (25). Collectively, these findings indicate that dysregulation of sialic acid metabolism plays a critical regulatory role in tumor progression and metastasis. This study examines the mechanistic foundation of sialic acid metabolism within tumor pathology by elucidating phenotypic changes and alterations in protein sialylation resulting from the exogenous administration of Neu5Ac in pertinent cellular models. This approach seeks to deepen our understanding of the underlying mechanisms and potentially identify novel therapeutic strategies for cancer treatment.

## 2 Methods

### 2.1 Clinical sample collection

Nine pairs of breast cancer tumors and nearby non-cancerous tissues were examined in this study, with samples taken from patients at Tianjin Cancer Hospital during 2020 to 2021.The protocol for the research project was approved by the Ethics Committee of Tianjin Medical University Cancer Institute and Hospital (Institutional Review Board: bc2019075). All participants provided written informed consent prior to sample collection, in accordance with the Declaration of Helsinki. The consent process specifically included permission for the use of biological samples for research.

### 2.2 Cell lines

Human breast cancer cell lines MDA-MB-231 and HCC1806 were utilised. Cells were procured from Zhejiang Bodi Biotechnology Co., Ltd. (Zhejiang, China). The culture medium for MDA-MB-231 cells was complete, containing 89% DMEM with high glucose, 10% fetal bovine serum (FBS), and 1% penicillin/streptomycin (PS).The complete medium for HCC1806 cells consisted of 89% RPMI-1640, 10% FBS, and 1% PS, and the cultures were incubated at 37°C in a humidified atmosphere containing 95% air and 5% CO_2_. Cell growth status was monitored daily; culture medium was refreshed as required to ensure optimal cell proliferation.

### 2.3 Drug treatment

During the logarithmic growth phase, cells were introduced into 6-well plates or 10 cm dishes. Following 24 hours, the medium was replaced with a fresh medium that included N-acetylneuraminic acid (Neu5Ac; TargetMol, T1638), with final concentrations set at 0, 0.5, and 1 mg/mL. Cells were treated continuously for 72 hours. Neu5Ac was stored at low temperature.

### 2.4 Cell proliferation assay

During the logarithmic growth phase, 3000 breast cancer cells were introduced into each well of 96-well plates. After seeding, at the 4h, 24h, 48h, 72h, 96h, and 120h time points, the existing medium was replaced with 100 µL of CCK-8 mixture (90% basal medium + 10% CCK-8 reagent). After a 1-hour incubation period, the plates were shaken for 30 seconds, and absorbance at 450 nm was recorded using a microplate reader. Data points representing cell number were collected on Days 0, 1, 2, 3, 4, and 5 for growth curve construction.

### 2.5 Wound healing assay

Cells from breast cancer in the logarithmic growth phase were seeded into 6-well plates and subjected to drug treatment. When confluence was reached, the medium was replaced by a serum-free medium (SFM) containing drugs for 12 hours. Subsequently, two linear, parallel wounds were created per well using a pipette tip positioned vertically against the plate edge. Images were captured at 0 h and 24 h post-wounding at four designated positions per well. Wound closure was analysed using ImageJ software.

### 2.6 Transwell invasion assay

Log-phase cells were serum-starved in SFM for 12 hours. Matrigel was diluted to 1 mg/mL in SFM and used to coat the transwell inserts. 500 µL of complete medium (10% FBS) was placed in the lower chambers of a 24-well plate. Serum-starved cells were digested, resuspended in SFM, adjusted to the required density, and 100 µL aliquots were seeded into the upper transwell chambers. Incubation of the plates occurred at 37°C with 5% CO_2_ for 48 hours. Inserts were then removed; medium, non-invaded cells, and matrigel were gently removed from the upper surface. Cells that invaded the lower surface were fixed using paraformaldehyde for 30 minutes, rinsed once with PBS, stained with 1 mL of crystal violet solution for 30 minutes, and then washed three times with PBS. Following air-drying, migrated cells were assessed qualitatively by microscopy, and quantitatively by counting cells in 3–5 random fields using ImageJ.

### 2.7 Cell cycle analysis

The cells were collected through trypsinisation, spun down, and the resulting pellets were resuspended in pre-cooled 70% ethanol for fixation for at least 2 hours at 4°C. Cells that were fixed underwent centrifugation, had ethanol removed, were washed with PBS, and then resuspended in a propidium iodide (PI) staining solution for 30 minutes at room temperature in the dark. Using flow cytometry, researchers analyzed cellular DNA content. The cell cycle phases (G0/G1, S, G2/M) were identified with FlowJo software to evaluate how sialic acid affects them.

### 2.8 Cell apoptosis detection

Double staining with Annexin V-APC/PI was performed. The harvested cells underwent washing, centrifugation, and were resuspended in Binding Buffer. As instructed by the kit, Annexin V-APC and PI were added, and the cells were incubated in the dark for 10 minutes. The apoptosis rates, including early and late stages, were measured using flow cytometry to determine the influence of sialic acid.

### 2.9 Gene expression analysis in tumour vs. normal tissue

The TCGA and GTEx databases provided publicly accessible data from breast cancer patients and normal breast tissue. GraphPad Prism was used to create boxplots comparing expression differences between cancerous and normal tissues to assess potential prognostic value in breast cancer.

### 2.10 Survival curve analysis

The study made use of the Kaplan-Meier Plotter online resource (https://kmplot.com/analysis/). To assess potential prognostic significance in breast cancer, gene names and survival data were used to produce Kaplan-Meier survival curves, analyzing the relationship between gene expression and patient survival rates.

### 2.11 Gene expression correlation analysis

The TIMER 3.0 online database (https://www.bioinformatics.com.cn/) was used. Target genes and genes of interest were input, selecting the relevant breast cancer dataset, to generate gene correlation plots.

### 2.12 mRNA library constructing and sequencing

Following extraction of cellular RNA, reverse transcription was performed, with subsequent quality assessment of the resulting cDNA. Libraries harboring distinct indices were then subjected to multiplexed pooling. Sequencing was conducted on the Illumina HiSeq, Illumina NovaSeq, or MGI2000 sequencing platform using the 2×150 bp paired-end (PE) sequencing mode, in accordance with the manufacturer’s instructions. Transcriptomic experiments and preliminary analyses were carried out by Genewiz Suzhou Biotechnology Co., Ltd.

### 2.13 Differential exon usage analysis

DEU analysis, which detects alterations in the rate of exon incorporation into transcripts (including aberrant splicing, altered transcription start sites, or polyadenylation sites affecting 5’ or 3’ boundary exon usage), was performed using the Bioconductor package DEXSeq (version 1.21.1).

### 2.14 GO, KEGG enrichment and GSEA analysis

The KEGG Compound database (http://www.kegg.jp/kegg/compound/) was used to annotate differentially expressed genes, and these annotations were later associated with the KEGG Pathway database (http://www.kegg.jp/kegg/pathway.html).

Gene Set Enrichment Analysis (GSEA) was conducted using the Python-based GSEApy package (version 1.1.4), implementing the GSEA algorithm. This analysis identified statistically significant enrichment of predefined gene sets based on differentially expressed genes derived from RNA sequencing data.

### 2.15 Metabolomics sample preparation and extraction

500 µL of an extraction solvent, composed of methanol and water in a 4:1 ratio and containing internal standards, was introduced to the cell pellets. Using a vortex, the suspensions were mixed vigorously for a duration of 3 minutes. The procedure involved subjecting the samples to three cycles of freezing and thawing, with each cycle comprising 5 minutes in liquid nitrogen, 5 minutes in dry ice, and thawing on ice with 2 minutes of vortexing. The extracts underwent centrifugation at 12,000 rpm for 10 minutes at 4°C, after which 300 µL of the supernatant was placed in a new tube and maintained at -20°C for 30 minutes. The tubes were subjected to a second round of centrifugation at 4°C, spinning at 12,000 rpm for 3 minutes, after which 200 µL of the clear supernatant was taken for LC-MS analysis.

Metabolomics was completed by Wuhan Metabo Biological Technology Co., Ltd.

### 2.16 Lectin flow cytometry

Following treatment, cells were harvested by trypsinisation to create single-cell suspensions. They underwent three washes with a washing buffer (PBS with 0.5% FBS) and were centrifuged at 600 rcf for 3 minutes. Cells were resuspended and incubated with lectins MAL-I or MAL-II for 1 hour at 4°C in the dark. Following three further washes, cells were incubated with APC-conjugated streptavidin (used as secondary reagent) for 1 hour at 4°C in the dark. After three final washes, cells were resuspended in buffer to an appropriate concentration for flow cytometry. Analysis and graphical representation were performed using FlowJo software.

### 2.17 Lectin pull-down

Cells that were treated were collected, rinsed, and broken down on ice with RIPA buffer containing protease inhibitors. The process involved sonication and centrifugation of lysates, followed by the collection of supernatants. Protein concentration was determined through a BCA assay. 500 µg of protein was incubated with 20 µg of biotinylated lectin overnight at 4°C. The combination of protein and lectin was left to incubate with streptavidin-linked agarose beads overnight at 4°C. The complexes underwent thorough washing with lysis/wash buffer using low-speed centrifugation. The bound proteins were eluted by heating at temperatures of 95°C or higher in SDS-PAGE loading buffer and then separated via SDS-PAGE. The gels were kept at 4°C for subsequent MS analysis.

### 2.18 Protein mass spectrometry

The protein bands of interest were cut out from gels stained with Coomassie. The gel fragments were then destained, underwent in-gel reduction and alkylation, and were washed. Trypsin was used for overnight in-gel digestion at 37°C. The peptides underwent extraction, desalting, concentration, and drying, and the dried samples were kept at -20°C before undergoing LC-MS/MS analysis.

### 2.19 Western blotting

Cells that were treated were collected, rinsed, and broken down on ice with RIPA buffer containing protease inhibitors. The lysates were sonicated, clarified by high-speed centrifugation at 4°C, and protein levels were measured using a BCA assay. The protein lysates were mixed with Laemmli sample buffer and heated at 95°C for 5 minutes to denature them. Proteins ranging from 10 to 25 µg were then separated using SDS-PAGE. The wet transfer method was used to transfer proteins onto PVDF membranes, which were then blocked with 5% skimmed milk powder for 60 minutes at room temperature. The primary antibody was used to incubate the membranes overnight at 4°C, with specific dilutions and antibody details outlined in the **Supplementary Information**. Membranes were treated with the correct HRP-conjugated secondary antibody for an hour at room temperature after being washed with TBST. Membranes underwent further washing and were then exposed to ECL chemiluminescent substrate for 60 seconds at room temperature, shielded from light. A chemiluminescence imaging system was used to capture the images. Band intensity was quantified using ImageJ software.

Antibody information is provided in the **Table 1**.

**Table 1:** Reagents and antibodies.

name	manufacturers	Product code	batch number	
FBS	Vivacell	C04001	2444605	
Penicillin-Streptomycin Solution	Vivacell	C3420-0100	2445428	
N-Acetylneuraminic acid	TargetMol	T1638		
CCK-8	Sparkjade	CT0001-A	FPHWL	
paraformaldehyde	Macklin	P804536	C17316030	
Crystal Violet Staining Solution	MeilunBio®	MA0148	Nov-14J	
transwell	Labselect	14341	30524089E	
Cell cycle kit	Beyotime Biotechnology	C1052	A227250522	
Apoptosis Assays Kit	Vazyme	A214-02	7E2813C5	
proteinase inhibitor	ABclonal	RM02916	9625606A20	
BCA	Sparkjade	EC0001-B	WTNKX	
Streptavidin Agarose	Beyotime Biotechnology	P2159	A306A250528	
Coomassie Brilliant Blue Staining Solution	Vazyme	E901-02	7E2952C5	
ECL Luminescent Solution	Vazyme	E423-01	7E1160A5	

antibody	manufacturers	Product code	batch number	dilution rate	
MAL-I	Vectorlabs	B-1315-2	ZL0828	1	2000
MAL-II	Vectorlabs	B-1265-1	ZL0809	1	1000
HIF-1A	HUABIO	HA722778	H651404035	1	4000
CCNB1	HUABIO	ET1608-27	H651264018	1	1000
Tubulin	proteintech	66031-1	10028345	1	5000
Goat Anti-Mouse IgG H&L (HRP)	Abcam	AB205719	1036603-1	1	10000
Goat Anti-Rabbit IgG H&L (HRP)	Abcam	AB205718	1036958-26	1	10000
APC-Streptavidin	ABclonal	RM02942	0000010226	1	20

### 2.20 Statistical analysis

Statistical analyses, including unpaired t-tests and one-way ANOVA, were performed using GraphPad Prism. Unless otherwise specified, P-values were calculated for all experimental procedures and analyses. A p-value of 0.05 or under was considered to indicate statistical significance. Data are expressed as mean ± standard deviation (SD), except where otherwise mentioned.

The platform at http://www.bioinformatics.com.cn/ pathway enrichment analysis for transcriptomic and proteomic datasets. The analysis and visualization of transcriptomic and metabolomic data, including the production of relevant figures, were conducted with support from Wuhan Metware Biotechnology Co., Ltd.

## 3 Result

### 3.1 UDP-GlcNAc levels showed significant upregulation within patient cancer tissues

**Figure 1:**
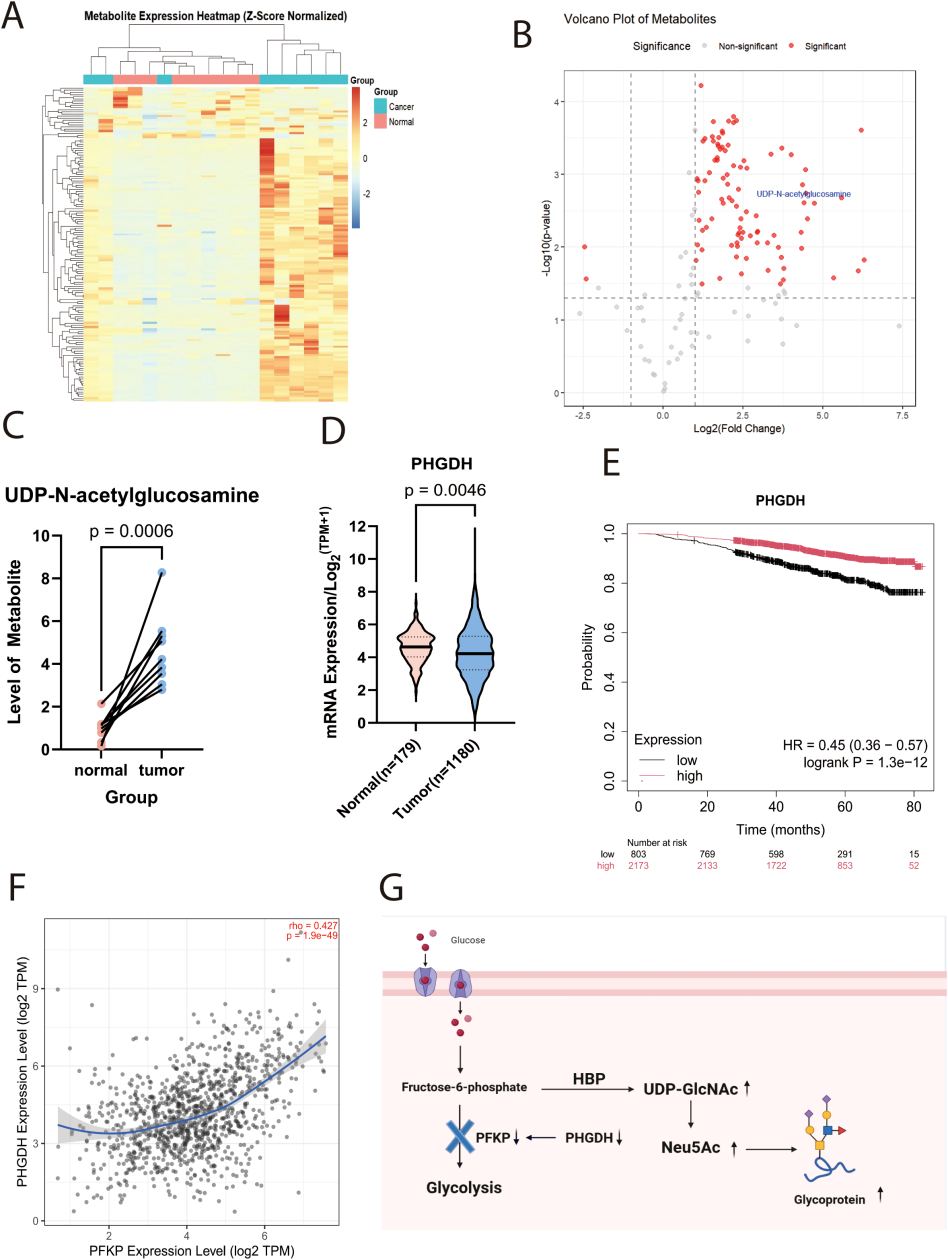
UDP-GlcNAc expression is upregulated in tumour tissue. **(A)** Heatmap of metabolomic profiling in patient tissues (n=9; Z-score standardised). **(B)** Volcano plot of metabolomic profiling in patient tissues. **(C)** Line chart depicting altered UDP-GlcNAc levels in tumour versus adjacent non-tumour tissues. **(D)** Box plots showingPHGDHmRNA expression levels in breast tissue versus breast carcinoma tissue from TCGA and GTEx databases. **(E)** Prognostic Kaplan-Meier curve showing overall survival of breast cancer patients in relation toPHGDHexpression. **(F)** Correlation analysis plot between PHGDH and PFKPexpression. **(G)** Schematic diagram illustrating the mechanism by which altered PHGDH expression affects UDP-GlcNAc levels.

Nine pairs of breast cancer tumors and nearby non-cancerous tissues were examined in this study, with samples taken from patients at Tianjin Cancer Hospital during 2020 to 2021. **Table 2** provides the pathological diagnoses for these patients. Compared to normal tissues, tumor tissues exhibited a marked increase in UDP-GlcNAc levels, as shown by metabolomic sequencing (**Figure 1A-C**,**Supplementary Table 1**).

**Table 2:** Clinical information of the patient.

serial number	age	ER(%)	PR(%)	HER2	KI67(%)	P53(%)	stages
1802501	49	1	1	0	50	90	T2N1
1802348	71	90	90	1	15	20	T2N0
1801077	53	80	90	2+	35	15	T2N0
1802508	44	80	10	0	40	70	T2N0
1800962	32	70	40	2+-3+	70	70	T2N0
1800961	79	90	20	1+	60	2	T2N0
1800855	71	90	90	2+	30	5	T2N3
1803507	59	90	80	0	70	2	T2N1
1800160	77	1	1	3+	40	1	T2N0
1800647	43	85	60	3+	30	30	T2N1

Supporting this hypothesis, research conducted by Matteo Rossi et al. demonstrated that the loss of Phosphoglycerate Dehydrogenase (PHGDH) expression leads to a reduction in PFK protein activity, particularly affecting the PFKP isoform. This reduction results in elevated intracellular levels of UDP-GlcNAc and its downstream metabolite, Neu5Ac (N-acetylneuraminic acid, the predominant sialic acid). Such an increase enhances protein sialylation, thereby conferring greater metastatic potential to cancer cells (18). Meanwhile, transcriptomic data analysis from the TCGA and GTEx projects confirmed that PHGDH expression is markedly downregulated in breast tumor tissues compared to the surrounding normal tissue (**Figure 1D**, **Supplementary Table 2**). Survival analysis further indicated that low PHGDH expression is strongly correlated with poor patient prognosis (Hazard Ratio [HR]=0.45; p<0.001) (**Figure 1E**). Importantly, we observed a significant positive correlation between PHGDH and PFKP expression at the transcriptomic level (**Figure 1F**).According to the preceding information, **Figure 1G** shows the schematic diagram of the mechanism.

Our previous research identified the specific expression of sialyl Lewis x (sLe^x^), a distinctive tetrasaccharide epitope terminating in sialic acid, on cell clusters in invasive mammary carcinoma exhibiting a micropapillary pattern. Importantly, the altered localization of sLe^x^ is significantly correlated with tumor metastasis and patient prognosis (26). Given the terminal position of sialic acid in the sLe^x^ structure and its crucial role in function (27), these findings collectively support the hypothesis that aberrant sialylation is a key factor in the pathogenesis and metastatic dissemination of breast cancer. Therefore, the main aim of this study is to explore how abnormal sialic acid modification contributes to the development and spread of breast cancer.

### 3.2 Exogenous sialic acid promotes cell invasion and metastasis

**Figure 2:**
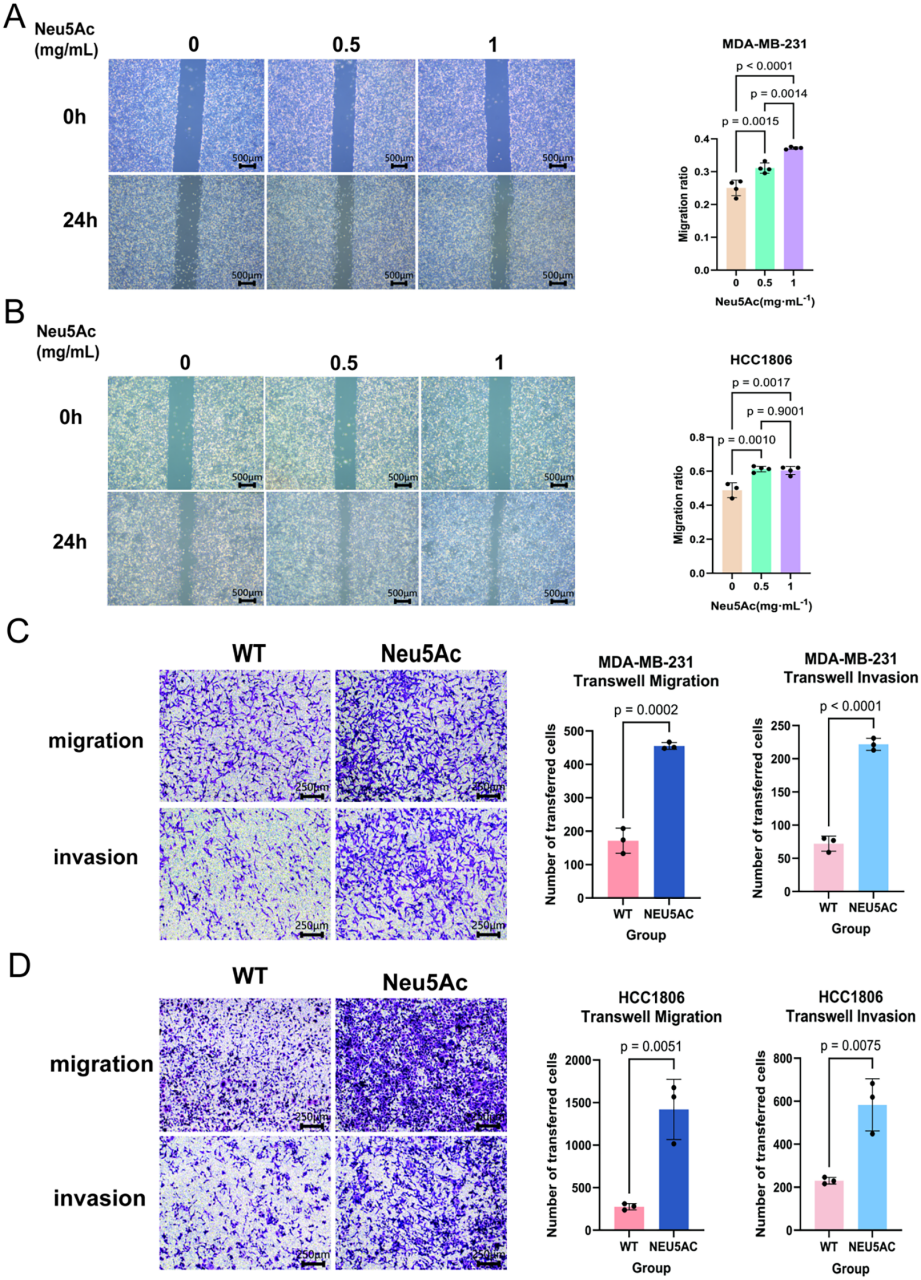
Neu5Ac supplementation enhances the invasive and migratory capacity of breast cancer cells following 72-hour treatment. **(A)** Wound healing (scratch) assay and quantitative analysis of MDA-MB-231 cells. **(B)** Wound healing (scratch) assay and quantitative analysis of HCC1806 cells. **(C)** Transwell migration assay and quantitative analysis of MDA-MB-231 cells. **(D)** Transwell migration assay and quantitative analysis of HCC1806 cells. Scale bar = 200 µm. Image analysis was performed using ImageJ. Error bars represent SD.

To elucidate the role of sialic acid in breast cancer, breast cancer cell lines were incubated for 72 hours in a medium supplemented with exogenous sialic acid (Neu5Ac). Experiments that followed aimed to examine its effects on the proliferation and migration of cells. Scratch wound healing assays (**Figures 2A, B**) demonstrated that Neu5Ac significantly promoted cell migration. Consistent with these observations, Neu5Ac was shown to improve the invasion and migration potential of breast cancer cells, as evidenced by Transwell migration and invasion assays (**Figures 2C, D**). In parallel, the influence of Neu5Ac on cell proliferation was tested with CCK-8 assays, which indicated that Neu5Ac did not have a significant impact on the proliferation of breast cancer cells (**Supplementary Figure 1A, B**). Further analysis of cell cycle progression and apoptosis via flow cytometry supported these findings (**Supplementary Figures 1C-E**). In summary, sialic acid greatly increases the migration and invasion potential of breast cancer cells.

### 3.3 Elevated sialic acid levels can modulate pathways related to cellular lipid metabolism.

**Figure 3:**
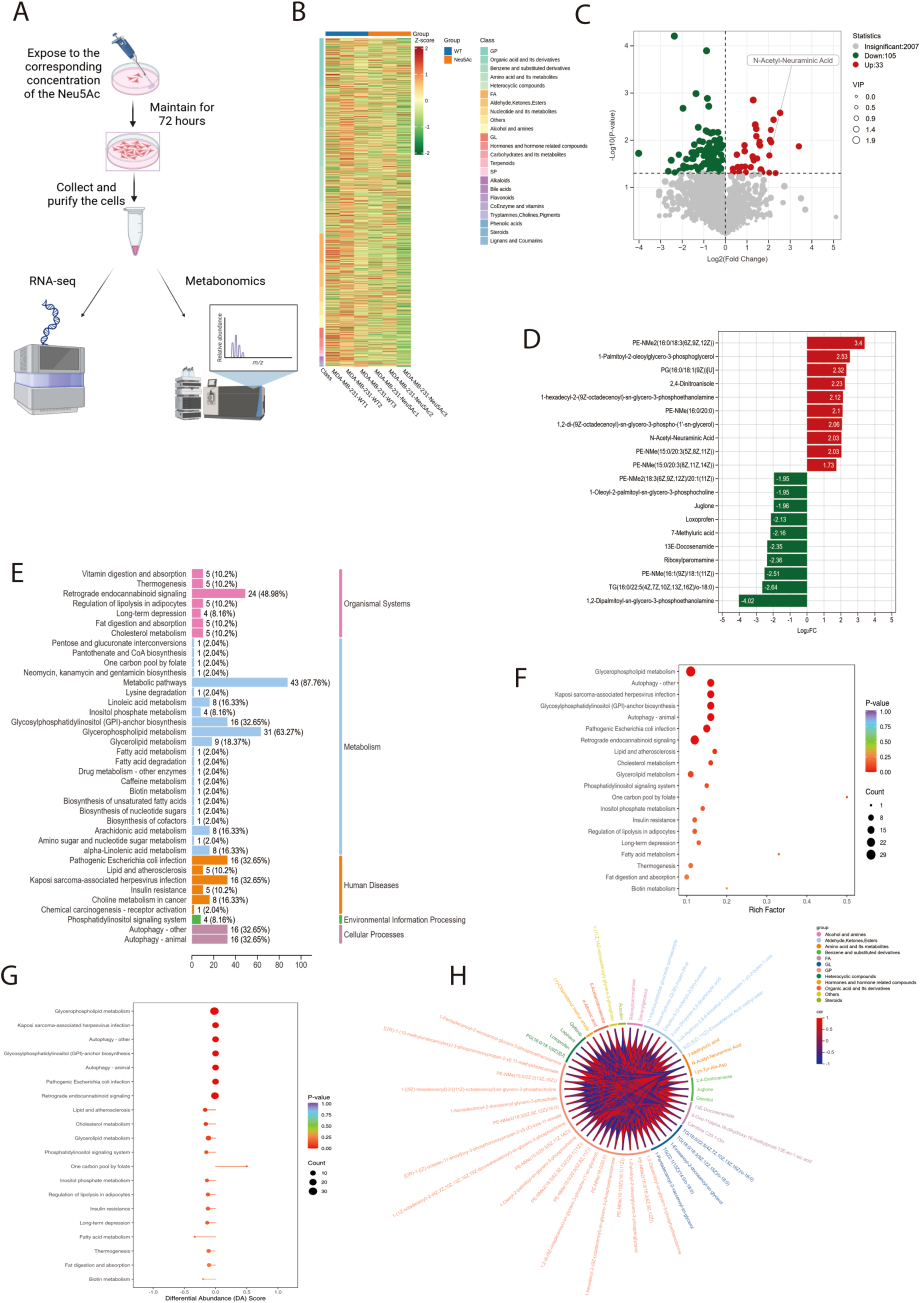
Neu5Ac treatment for 72 hours induces metabolic reprogramming in breast cancer cells. **(A)** Experimental workflow: Cells were collected for multi-omics analysis after 72-hour Neu5Ac treatment in culture medium. **(B)** Clustering analysis of differentially abundant metabolites identified in the metabolomic screen. **(C)** Volcano plot of differentially abundant metabolites (Green: Downregulated; Red: Upregulated). **(D)** Bar chart showing the top 10 upregulated and downregulated differentially abundant metabolites by fold change. **(E)** Pathway classification of differentially abundant metabolites. Y-axis: Metabolic pathway names; X-axis: Number of significant metabolites per pathway and the proportion (%) relative to total assigned metabolites. **(F)** KEGG pathway enrichment analysis of differentially abundant metabolites. Rich Factor represents the ratio of significant metabolites to total metabolites annotated to a pathway. **(G)** Overall pathway analysis using Differential Abundance Score (DAS). An X-axis score of 1 indicates that all detected metabolites within a pathway trend upwards; -1 indicates all trend downwards. **(H)**Chord diagram depicting correlation coefficients between differentially abundant metabolites (Red: Positive Pearson correlation; Blue: Negative Pearson correlation).

To elucidate the mechanism by which Neu5Ac facilitates breast cancer cell migration and invasion, we conducted untargeted metabolomic profiling on breast cancer cell lines treated with Neu5Ac (**Figure 3A**). The untargeted metabolomics analysis identified approximately 2,500 metabolites, with organic acids and their derivatives (15.4%), amino acids and their derivatives (8.59%), glycerophospholipids (12.9%), and fatty acids (10.7%) being the predominant classes (**Supplementary Table 3**). Based on these results, we constructed a clustered heatmap of the metabolites (**Figure 3B**). A volcano plot depicting the differential metabolites between the control and Neu5Ac-treated groups revealed a total of 138 significantly altered metabolites, of which 105 were downregulated and 33 were upregulated (**Figure 3C**). Notably, intracellular Neu5Ac levels were significantly elevated following treatment. This finding indicates that exogenous administration of Neu5Ac effectively replicates the upregulation of Neu5Ac observed in tumor tissues, thereby validating this model for investigating the role of Neu5Ac in cancer. Bar charts illustrating the top ten upregulated and downregulated metabolites identified several members of the phospholipid family, along with 13E-Docosenamide, a specific long-chain unsaturated fatty acid amide, as undergoing significant alterations (**Figure 3D**). This observation strongly suggests the modulation of associated lipid metabolic pathways. Pathway enrichment analysis of the metabolomic dataset revealed a downregulation in several pathways related to cellular lipid metabolism (**Figures 3E-H**), including glycerolipid and fatty acid metabolism.

In conclusion, our findings indicate that exogenous Neu5Ac supplementation induces metabolic reprogramming in breast cancer cells, with particularly pronounced effects on pathways related to lipid metabolism.

### 3.4 Alterations in cellular transcription were observed upon the exogenous addition of sialic acid

**Figure 4:**
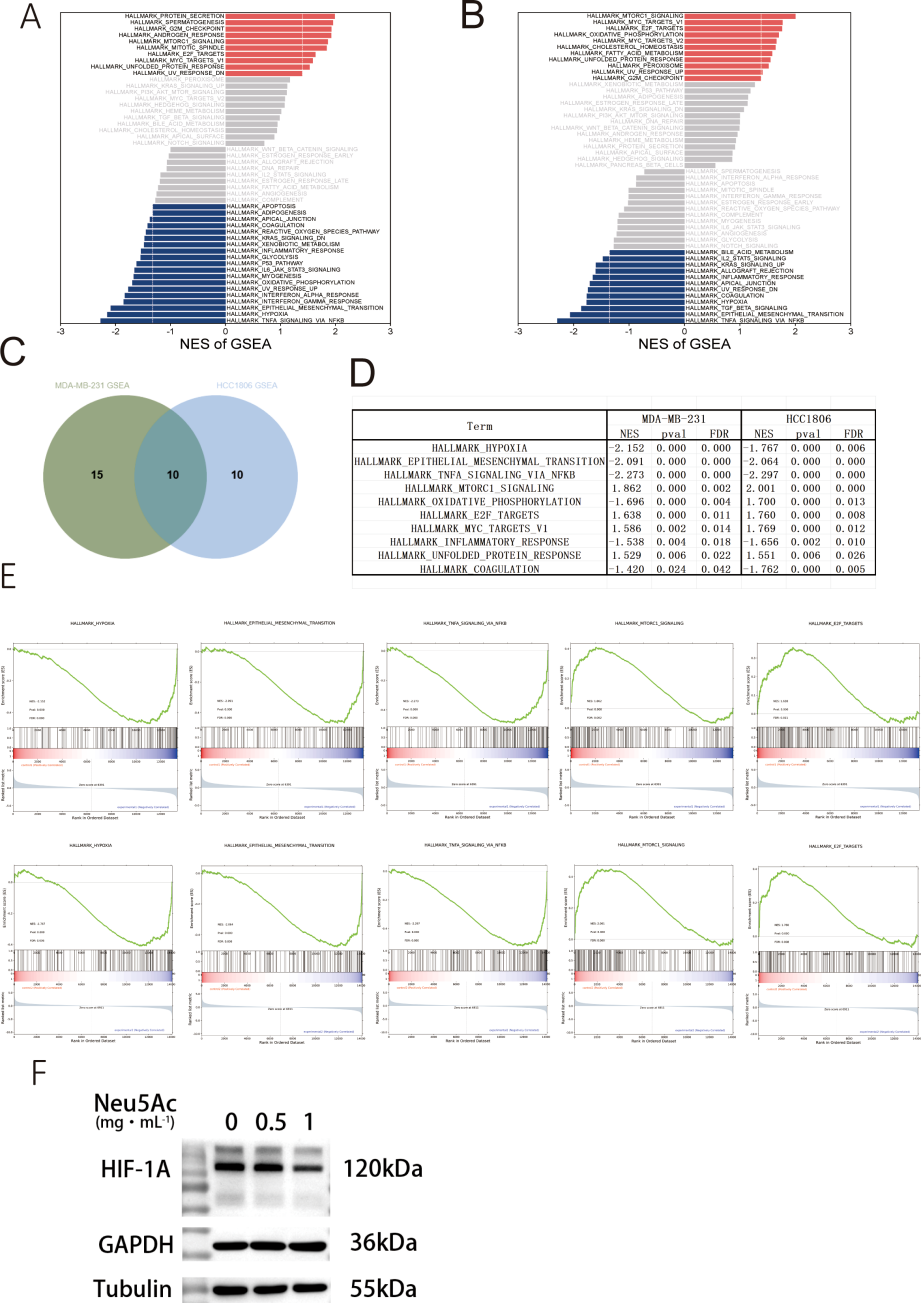
Neu5Ac treatment for 72 hours induces transcriptomic alterations in breast cancer cells. **(A)** Gene Set Enrichment Analysis (GSEA) of MDA-MB-231 RNA-seq data (Red: Upregulated pathways; Blue: Downregulated pathways). **(B)** Gene Set Enrichment Analysis (GSEA) of HCC1806 RNA-seq data. **(C)** Venn diagram showing 10 common pathways significantly altered in both MDA-MB-231 and HCC1806 cells. **(D)** Details of common differentially regulated pathways. **(E)** Downregulated pathways include Hypoxia, Epithelial-Mesenchymal Transition (EMT), and TNFα signaling; Upregulated pathways include E2F Targets and mTORC1 signaling.(F&G)Representative western blots validating protein expression. **(F)** Hypoxia-related factor HIF-1α.

To elucidate the mechanisms driving phenotypic and metabolic alterations in cells following exogenous sialic acid supplementation, the MDA-MB-231 and HCC1806 breast cancer cell lines underwent transcriptomic sequencing (**Supplementary Table 4**). Complementary data (**Supplementary Figures 2A, B**) suggest that the addition of exogenous sialic acid did not lead to changes in RNA regulatory processes such as exon skipping or alternative splicing. KEGG and GO analysis of genes with significant differential expression (p < 0.05) in both MDA-MB-231 and HCC1806 cells revealed an enrichment of pathways pertaining to the endoplasmic reticulum (ER)-to-Golgi transport (**Supplementary Figures 2C-F**). It is noteworthy that cellular sialic acid modification also takes place within the ER and Golgi apparatus. Further examination through Hallmark pathway analysis and GSEA (**Figures 4A, B**, **Supplementary Table 5**) identified 25 and 20 pathways that were significantly dysregulated in MDA-MB-231 and HCC1806 cells, respectively. Considering the inherent variability in cellular gene expression regulation, we examined these dysregulated pathways for commonalities between the two cell lines. This comparative analysis identified 10 intersecting pathways (**Figure 4C**).Alterations in the oxidative phosphorylation pathway exhibited variability between the cell lines (**Figure 4D**) and were consequently excluded from further analysis. Interestingly, well-established pro-tumorigenic pathways commonly associated with cancer, such as hypoxia signaling, epithelial-to-mesenchymal transition (EMT), and tumor necrosis factor alpha (TNF-α) signaling, were found to be suppressed. In contrast, pathways related to E2F cell cycle targets, MYC signaling, energy metabolism regulation via mTORC1 signaling, and the unfolded protein response (UPR) within the endoplasmic reticulum were upregulated (**Figure 4E**). Immunoblot analysis of total cellular protein extracts (**Figure 4F**)revealed a significant reduction in HIF-1α protein levels which is hypoxia-inducible factor (28).

**Figure 5:**
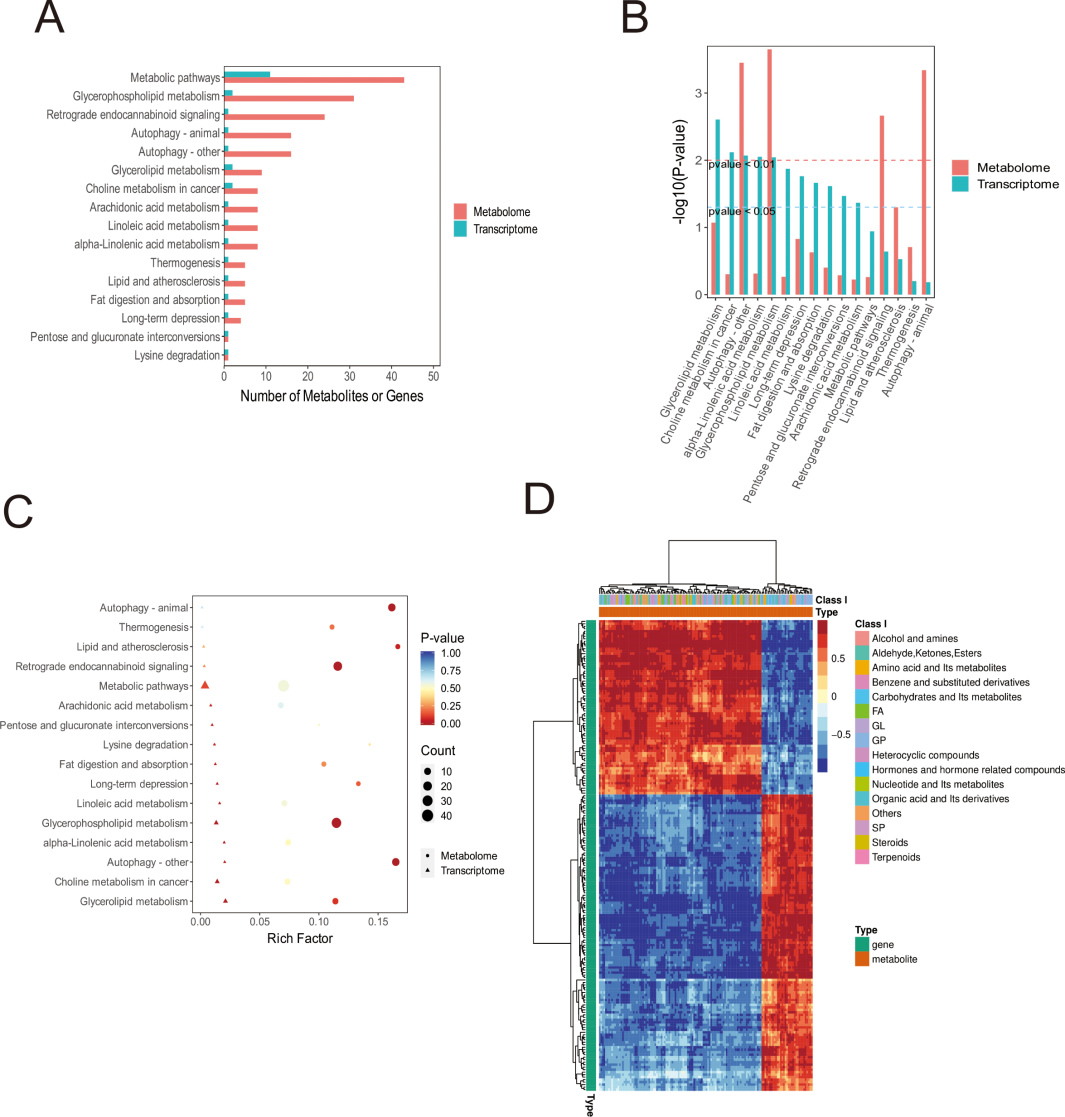
Integrated Transcriptomic and Metabolomic Analysis of MDA-MB-231 Cells. **(A)** Bar chart displaying co-enriched KEGG pathways. Number of DEGs and metabolites enriched per pathway are indicated. **(B)** Bar chart depicting the significance (-log10(p-value)) of DEG/metabolite co-enriched KEGG pathways. **(C)** Bubble plot visualising co-enriched KEGG pathways. Dimensions include pathway coordinates (X/Y), size and colour of bubbles based on enrichment significance, and shape denoting pathway origin/directionality. **(D)** Hierarchical clustering heatmap of correlations between all DEGs and differentially abundant metabolites (Red: Positive Pearson correlation; Blue: Negative Pearson correlation). Rows represent genes; Columns represent metabolites.

Subsequent integrated analysis of transcriptomic and metabolomic datasets for MDA-MB-231 cells indicated that the most substantial numerical changes in RNA and metabolite levels were observed within metabolic pathways, including glycerophospholipid metabolism, autophagy, glycerolipid metabolism, and choline metabolism in cancer (**Figure 5A**). Importantly, statistically significant changes at both the transcriptomic and metabolomic levels were confirmed exclusively for glycerophospholipid metabolism and autophagy pathways (**Figure 5B**). Overall, these integrated data do not completely explain the observed phenotypic and metabolic changes in the cells. This discrepancy may arise from the primary role of sialic acid in mediating cellular effects through post-translational modification (PTM) of proteins, a mechanism that is potentially less likely to induce substantial transcriptional changes. Nonetheless, our findings provide sufficient evidence that exogenous sialic acid reprograms the metabolism of breast cancer cells, leading to significant alterations in glycerophospholipid metabolism, fatty acid metabolism, and related pathways(**Figures 5C, D**).

### 3.5 Sialylation modifications modulate lipid metabolism and transport-related proteins

**Figure 6:**
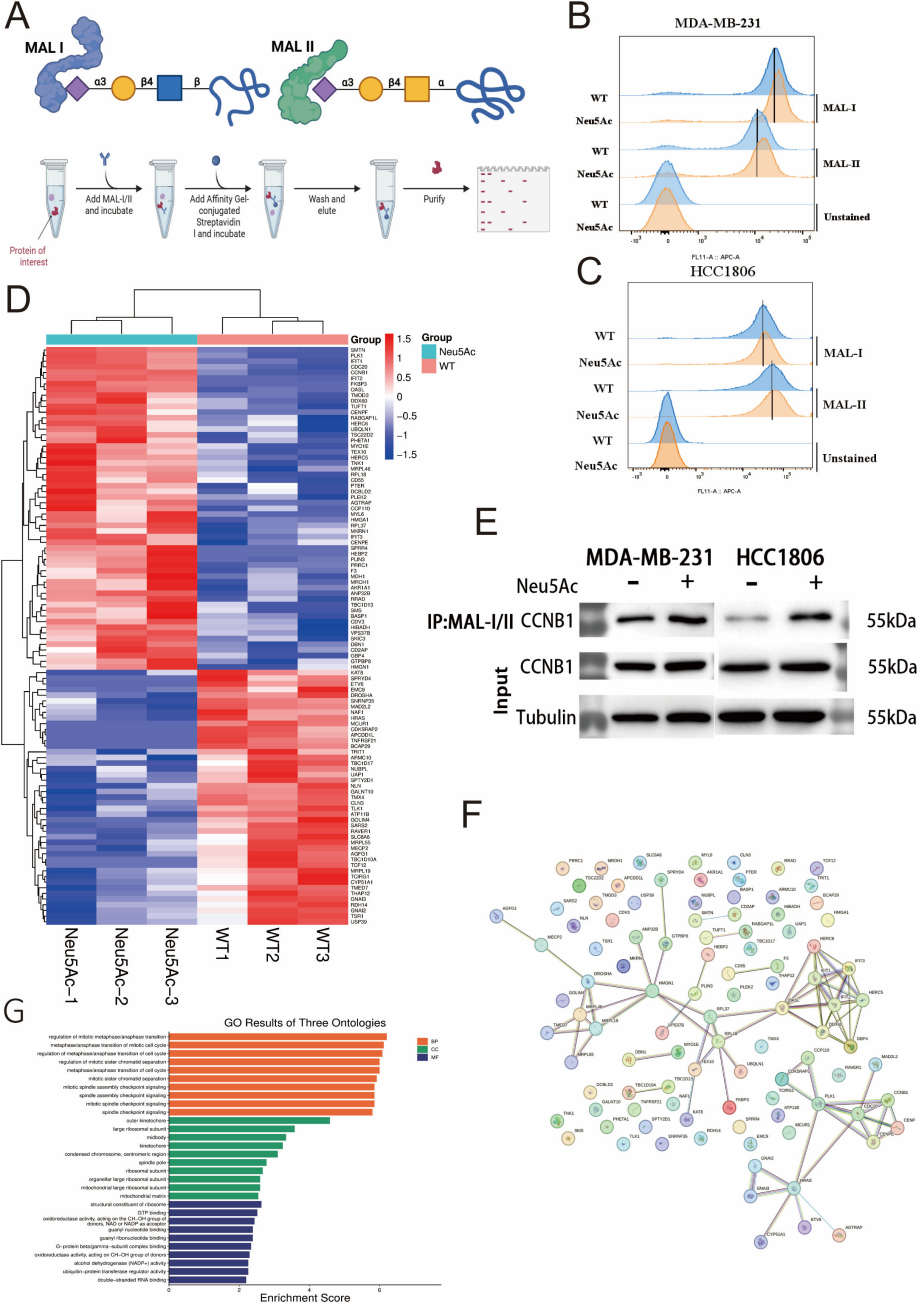
α-2,3-Sialylation Proteomics. **(A)** Schematic of MAL-I/MAL-II lectin co-immunoprecipitation workflow. **(B, C)** Increased α-2,3-sialylation levels detected via lectin blotting in Neu5Ac-treated **(B)** MDA-MB-231 and **(C)** HCC1806 cells. **(D)** Clustered heatmap of differentially expressed α-2,3-sialylated proteins in the proteomic screen (Red: Upregulated; Blue: Downregulated). **(E)** Validation of increased CCNB1 sialylation levels by lectin co-immunoprecipitation. **(F)** Protein-protein interaction network of proteins exhibiting differential α-2,3-sialylation. **(G)** Pathway enrichment analysis of differentially sialylated proteins.

To further elucidate the impact of Neu5Ac upregulation on sialylation, we utilized glycan-specific lectins, MAL-I and MAL-II(**Figure 6A**, **Supplymentary Figures 3A, B**), as probes to detect α-2,3-linked sialic acid residues (29, 30). Flow cytometry analysis demonstrated a rightward shift in the peak signal intensity for α-2,3-sialic acid in both MDA-MB-231 and HCC1806 cells 24 hours post-Neu5Ac treatment (**Figures 6B, C**). This shift suggests an increase in α-2,3-linked sialic acid modifications within these cell lines.

This finding aligns with the observations of Chen (16), which proposed a potential link between elevated cellular sialic acid levels and enhanced migratory capacity. To identify proteins whose sialylation status was altered by Neu5Ac upregulation, we initially employed lectin-mediated precipitation of sialylated proteins. The enriched fraction was subsequently analyzed using mass spectrometry (MS) (**Figure 6A**). Comparative MS analysis identified 6104 proteins in both experimental and control groups (**Supplementary Table 6**). By applying stringent criteria (p-value < 0.05, fold-change > 1.5), we identified 102 proteins with significant alterations in sialylation: 57 were upregulated and 45 were downregulated (**Supplementary Table 7**, **Figure 6D**).Alterations in sialylation have the potential to affect protein phosphorylation and, consequently, protein function (19). Given the metabolomic data indicating possible dysregulation of lipid metabolism, our investigation concentrated on differentially sialylated proteins associated with lipid metabolism. Notably, sialylation was found to be increased on PLIN3, a regulator of lipid storage (31), while it was decreased on CYP51A1, which is involved in cholesterol biosynthesis (32), and ATP11B, which participates in membrane lipid transport and metabolism (33). Through exogenous addition of sialic acid, we observed a significant enhancement in cellular migration and invasive potential. Notably, cyclin B1 (CCNB1), which has been reported as a key gene implicated in breast cancer brain metastasis (34),was also found to exhibit elevated sialylation levels in our Western blotting experiments. Subsequent *in vitro* Western blot analysis confirmed an increased level of sialylation on the CCNB1 protein, suggesting that this modification may contribute to its pro-metastatic function (**Figure 6E**).Our simultaneous analysis of protein-protein interaction networks (**Figure 6F**) and pathway enrichment (**Figure 6G**) on other differentially expressed proteins offers initial evidence that protein sialylation is involved in various pathways.

## 4 Discussion

Glycosylation involves a range of modifications, including sialylation, fucosylation, and mannosylation. These modifications are classified into N-glycans and O-glycans, depending on the specific amino acid to which the initial sugar molecule is attached (35). As such, glycosylation constitutes a complex and functionally diverse form of post-translational modification. The present study focuses on sialylation, a modification commonly found at the terminal positions of both N- and O-glycans. This unique positioning endows sialylation with a crucial role in processes such as intercellular recognition, signal transduction, and the regulation of the immune microenvironment (36).

The study began with an analysis of metabolomic data obtained from breast cancer patient tissue samples, revealing a significant upregulation of UDP-GlcNAc levels in breast tumor tissues. Fructose-6-phosphate, a glucose metabolite, can either enter the glycolytic pathway via fructokinase catalysis or serve as a precursor in the hexosamine biosynthetic pathway (HBP) to produce UDP-GlcNAc. The latter acts as a precursor for the biosynthesis of Neu5Ac, the substrate for sialylation (37). Consequently, it is hypothesized that within tumor tissues, there is either a suppression of glycolytic flux or an aberrant activation of the hexosamine biosynthetic pathway (HBP). Research conducted by Matteo Rossi (18) demonstrated that phosphoglycerate dehydrogenase (PHGDH) significantly reduces phosphofructokinase platelet (PFKP) activity, thereby inhibiting glycolysis and enhancing the synthesis of UDP-N-acetylglucosamine (UDP-GlcNAc) and its downstream metabolite, N-acetylneuraminic acid (Neu5Ac). Our bioinformatic analysis revealed a marked reduction in PHGDH expression in tumor tissues, which shows a strong positive correlation with PFKP expression. Survival analysis further indicated that low PHGDH expression is significantly associated with reduced patient survival. Collectively, these observations suggest that glycolytic suppression or aberrant activation of the HBP may be a critical aspect of tumor metabolic reprogramming.

The significant elevation of UDP-GlcNAc in breast cancer tissues likely exerts a broader influence by altering the global glycosylation landscape. As UDP-GlcNAc serves as the core donor for cellular glycosylation, its accumulation has the potential to remodel the tumor cell glycocalyx (8). Such altered expression could impact the metabolism of various sugar moieties, including fucose, mannose, and sialic acid. Building upon previous research conducted by our group, which identified the specific expression of sialyl Lewis X (sLex) glycan chains on the surface of tumor cell clusters within invasive micropapillary carcinoma (IMPC) of the breast, we observed that the altered localization of these chains was correlated with patient prognosis (26). IMPC is recognized for its highly aggressive and metastatic characteristics, typified by cohort-based invasion (38). Consequently, our current study aims to elucidate the relationship between this distinct invasive capability and glycosylation. Considering that sialic acid constitutes the terminal sugar of the sLex chain and its synthesis is directly dependent on the availability of UDP-GlcNAc, the observed upregulation of UDP-GlcNAc may facilitate the formation of various sialylated glycans. This could potentially enhance overall cellular sialylation, thereby influencing tumor invasion and metastatic potential.

To clarify the role of sialic acid in breast cancer metastasis, we utilized a model system involving the exogenous addition of Neu5Ac to cell culture media. Both wound healing and transwell assays indicated that Neu5Ac treatment significantly augmented the migratory and invasive capacities of MDA-MB-231 and HCC1806 cell lines. We observed that the scratch wound healing of MDA-MB-231 cells exhibits a dose-dependent relationship with the concentration of Neu5Ac. However, this phenomenon is not observed in HCC1806 cells. We propose that the enhancement of cell motility induced by Neu5Ac is limited, with no further change likely occurring beyond a certain threshold. Importantly, this pro-metastatic effect was independent of cell proliferation, cell cycle progression, or apoptosis, indicating that sialic acid may influence cellular motility through an alternative mechanism. To elucidate the underlying mechanisms, we conducted transcriptomic and metabolomic profiling on treated cells.

Metabolomic analysis revealed a substantial increase in intracellular Neu5Ac levels, thereby confirming the efficacy of our exogenous treatment model. Furthermore, 138 metabolites demonstrated significant alterations. Classification based on primary metabolite class indicated that lipids constituted 45% (62/138) of the altered metabolites. Notably, numerous glycerophospholipids and sphingomyelins were significantly downregulated, along with 7-methyluric acid from purine metabolism. Pathway enrichment analysis further underscored significant downregulation of lipid-related pathways, including glycerolipid metabolism and fatty acid metabolism. This extensive remodeling of lipid metabolism may affect properties such as membrane fluidity, signaling lipid generation, or lipid raft composition, ultimately impairing cell migration. Enrichment was also observed in pathways associated with autophagy. However, subsequent apoptosis assays conducted on Neu5Ac-supplemented cells demonstrated no significant differences.

An integrated analysis of transcriptomic and metabolomic data elucidated the intricate effects of sialic acid. KEGG pathway analysis demonstrated enrichment in the endoplasmic reticulum-Golgi transport pathway, aligning with glycosylation site involvement; however, overall transcriptional changes were modest, with only ten common pathways showing significant alterations across both datasets. This disparity between substantial metabolic changes and limited transcriptional modifications suggests that the effects of sialic acid predominantly occur at the post-translational level, exerting minimal influence on transcriptional pathways. Subsequent validation experiments indicated a significant reduction in the protein levels of the hypoxia-inducible transcription factor HIF-1A. This finding implies that sialic acid may indirectly modulate downstream gene expression, potentially through the destabilization of HIF-1A protein or by reducing its translational efficiency. Specifically, the downregulation of HIF-1α protein expression under normoxic conditions may contribute to the lipid metabolic alterations observed. Previous research has established a close association between HIF-1α expression and cellular glycolytic and lipid metabolism (39). The attenuation of HIF-1α expression can result in the downregulation of lipid metabolic pathways, consequently influencing tumor cell behavior. HIF-1α plays a critical role in regulating fatty acid uptake and storage within tumor cells. Research indicates that HIF-1α induces the expression of fatty acid-binding proteins, such as FABP3 and FABP7, which facilitate fatty acid uptake and lipid droplet formation—processes essential for cell growth and survival during hypoxia-reoxygenation cycles (40). In the context of hepatocellular carcinoma, reduced expression of HIF-1α is associated with diminished lipid metabolic reprogramming, partly due to the suppression of key metabolic enzymes (41). Consequently, the administration of exogenous Neu5Ac, which leads to decreased HIF-1α levels under normoxic conditions, may impair fatty acid uptake and storage, thereby compromising the metabolic adaptability of tumor cells.

To elucidate the molecular mechanisms underlying sialic acid-mediated metastasis, we employed lectin affinity purification in conjunction with mass spectrometry to systematically identify proteins with altered sialylation status following Neu5Ac treatment. This analysis revealed 57 upregulated and 45 downregulated sialylated proteins. The integration of metabolomic and transcriptomic data has elucidated substantial metabolic reprogramming, notably characterized by the downregulation of lipid pathways, such as glycerolipid and fatty acid metabolism. This observation aligns closely with the altered sialylation status of key lipid metabolic proteins, including PLIN3, CYP51A1, and ATP11B, as identified through mass spectrometry. Sialylation may potentially influence the conformation, stability, subcellular localization, or interactions of these proteins, thereby disrupting critical processes such as lipid droplet formation, cholesterol synthesis, and membrane lipid transport (42–44). In CHEN’s research, The MAA(MAL-II) signal of the cells has changed, but the SNA signal has not shown significant differences. Based on his research, we did not verify the SNA (16). Concurrent analyses using lectin flow cytometry and sialylated proteomics have corroborated that exogenous sialic acid not only significantly increases global surface α-2,3-linked sialic acid levels (45) but also, more critically, induces the sialylation of specific proteins CCNB1. Notably, CCNB1 (Cyclin B1) is a pivotal regulator of the cell cycle, and its aberrant expression has been associated with increased tumor invasiveness (46). The findings suggest that the pro-metastatic effects of sialylation are likely mediated not by a single mechanism but through a coordinated impact on a variety of functional proteins, including those involved in lipid metabolism and cell cycle regulation, collectively contributing to a pro-metastatic cellular phenotype. Our sialylome analysis revealed an enrichment not only of membrane and secreted proteins, which are traditionally considered primary sites of sialylation (47, 48), but also of intracellular proteins, notably metabolic enzymes and cell cycle regulators. The sialylation of these intracellular proteins may influence intracellular signaling pathways, metabolic regulation, and cell cycle progression through mechanisms that remain to be elucidated, thereby facilitating tumor cell invasion and metastasis through multiple pathways. This discovery provides a novel perspective on the complex role of sialic acid in cancer and represents a significant advancement in the field.

However, the study has inherent limitations that necessitate further investigation. While elevated levels of UDP-GlcNAc were detected in clinical breast cancer tissue samples, the limited availability of tissue samples precluded the direct measurement of Neu5Ac levels and the analysis of specific protein sialylation statuses within the corresponding patient cohort. It is important to acknowledge that UDP-GlcNAc is not exclusive to breast tissue, as it is expressed across various tissues and may exhibit similar dysregulation in other cancer types. Nevertheless, our findings offer significant insights into the role of sialic acid in tumor metastasis, warranting validation through future multi-center clinical studies. Breast cancer exhibits considerable heterogeneity with multiple distinct molecular subtypes in the clinical setting, and significant differences can exist between these subtypes (49). The MDA-MB-231 and HCC1806 cell lines used in this study both represent highly migratory models of triple-negative breast cancer (TNBC) (50). Classical luminal breast cancer cell lines, including T-47D, ZR-75-1, and BT474, exhibit relatively weaker *in vitro* proliferative and migratory capacities compared to triple-negative breast cancer cell lines (51). Consequently, they were not selected as experimental subjects in the present study. Future studies should therefore include a broader panel of breast cancer cell lines encompassing the diversity of molecular subtypes. Additionally, this study, guided by our previous findings, primarily concentrated on α-2,3-sialic acid modifications, potentially neglecting the contributions of other sialic acid linkage types. Future research should expand its focus to include a variety of sialylation types to achieve a comprehensive understanding of their roles in tumor metastasis. Existing literature indicates that different sialic acid linkages have distinct functions in cancer cells (52), with α-2,6-linked sialic acid playing a unique role in tumor cell invasion and lung metastasis (16). Future research endeavors should undertake a systematic comparison of the effects of α-2,3-, α-2,6-, and α-2,8-sialylation across various tumor contexts to elucidate their distinct mechanisms of action. Such insights have the potential to guide the development of more precise targeted therapeutic strategies. Furthermore, the incorporation of multi-omics approaches will enhance the understanding of the systemic impacts of sialylation on intracellular signaling networks.

## 5 Conclusion

This study primarily investigates the pivotal role and molecular mechanisms of Sialic Acid (Neu5Ac) in the metastasis of breast cancer. Initial metabolomic analyses of clinical tissue samples indicated an upregulation of UDP-GlcNAc, a direct precursor of Neu5Ac. This upregulation potentially facilitates the formation of sialylated glycans, thereby increasing overall cellular sialic acid levels and enhancing the invasive and metastatic potential of tumors. Experimental validation through cellular phenotypic assays demonstrated that exogenous supplementation with Neu5Ac significantly augmented the migratory and invasive capacities of MDA-MB-231 and HCC1806 cell lines. Importantly, this effect was observed to be independent of changes in cell proliferation, cell cycle progression, or apoptosis, suggesting that Neu5Ac modulates cellular motility through a distinct mechanism. Metabolomic analyses confirmed the efficient intracellular uptake of Neu5Ac and revealed its role in inducing significant metabolic reprogramming. Notably, transcriptomic analysis did not reveal any comparable significant changes. This distinct dissociation between transcriptional and metabolic responses strongly suggests that Neu5Ac primarily influences cellular processes through post-translational modifications (PTMs) rather than transcriptional regulation. Additionally, our results indicate that Neu5Ac treatment leads to a reduction in HIF-1α protein levels without corresponding changes at the transcriptional level, suggesting post-transcriptional regulation, such as effects on protein stability or translation, as the mechanism through which Neu5Ac exerts its influence on HIF-1α. The downregulation of HIF-1α may subsequently affect the expression of its target genes, many of which are involved in metabolic pathways, such as lipid metabolism, thereby indirectly contributing to the overall effects of Neu5Ac.In conclusion, the integration of flow cytometry and proteomic analyses has substantiated that Neu5Ac globally enhances α-2,3-linked sialylation levels and specifically modulates proteins associated with metastasis, such as CCNB1. Sialic acid enrichment analysis not only identified the anticipated membrane-bound and secreted proteins but also uncovered a significant involvement of intracellular proteins, including cyclins and metabolic enzymes. Future research should aim to conduct a more comprehensive analysis of sialylation types and linkages, employ integrated multi-omics approaches to elucidate the global mechanisms through which sialic acid influences signaling networks, and pursue multi-center clinical validation. This study lays the groundwork for the development of precision-targeted therapeutic strategies against metastasis.

## Ethical approval

The protocol for the research project was approved by the Ethics Committee of Tianjin Medical University Cancer Institute and Hospital (Institutional Review Board: bc2019075). All participants provided written informed consent prior to sample collection, in accordance with the Declaration of Helsinki. The consent process specifically included permission for the use of biological samples for research.

## Data Availability

The datasets presented in this study can be found in online repositories. The names of the repository/repositories and accession number(s) can be found in the article/**Supplementary Material**.

## Supplementary material

Supplementary Table 1 The metabolic data of the organization.

Supplementary Table 2 PHGDH data summarizing.

Supplementary Table 3 Add the data of the cellular metabolic profile of the Neu5Ac.

Supplementary Table 4 Add the data of the cellular RNA sequence of the Neu5Ac.

Supplementary Table 5 The data of GSEA in RNA sequence.

Supplementary Table 6 The data of α,2-3-Sialylation Proteomics.

Supplementary Table 7 Analysis of differential expression of sialylated protein groupsAnalysis of differential expression of sialylated protein groups.
