# Supplementary figures and images for: Aberrant sialic acid metabolism promotes metabolic reprogramming and metastasis in breast cancer
Source: Front Oncol. 2025 Nov 17;15:1698087. doi: 10.3389/fonc.2025.1698087 (PMC12665555; doi:10.3389/fonc.2025.1698087)

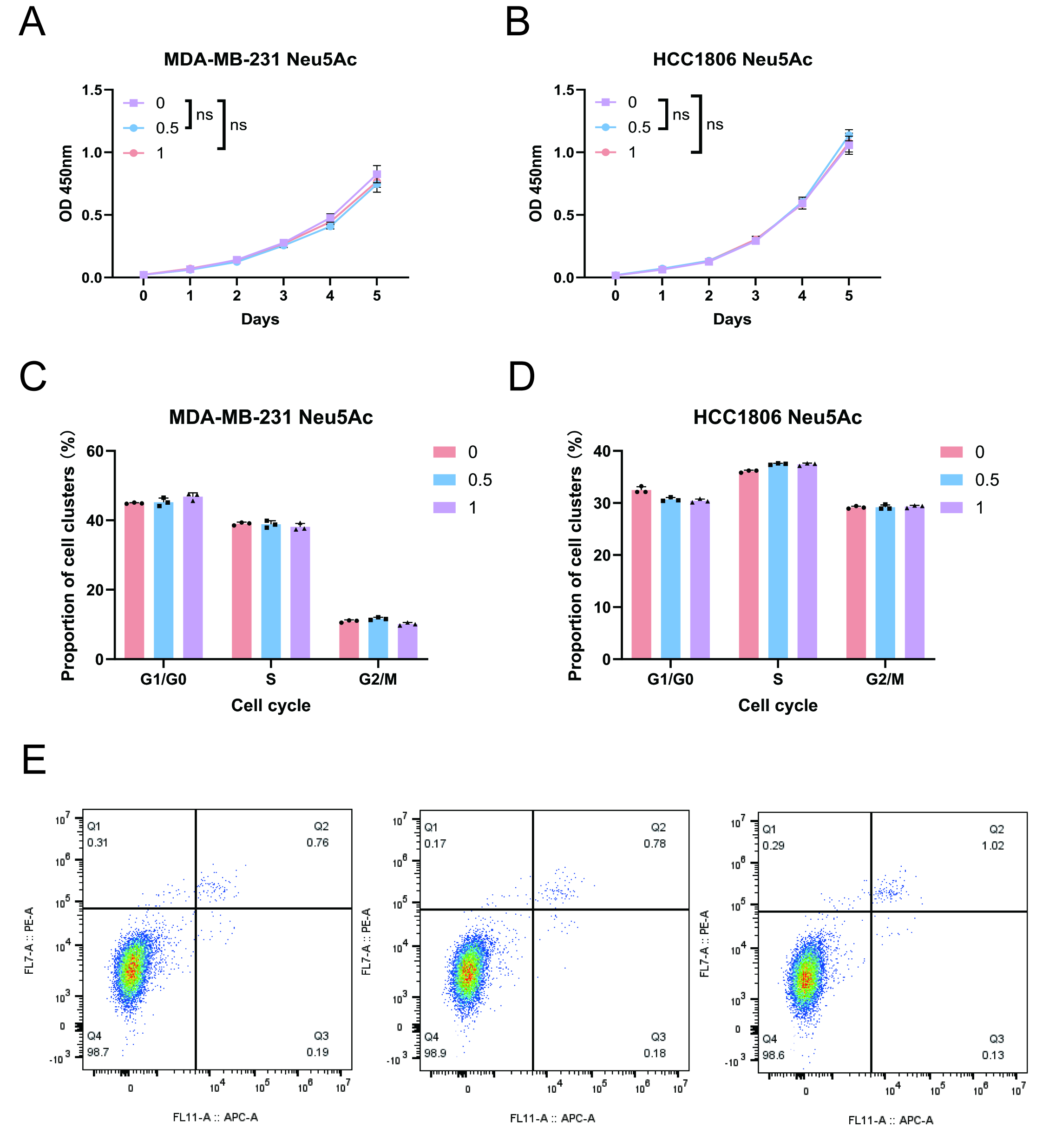

Supplement: Supplementary file 8 [file Image1.tif]

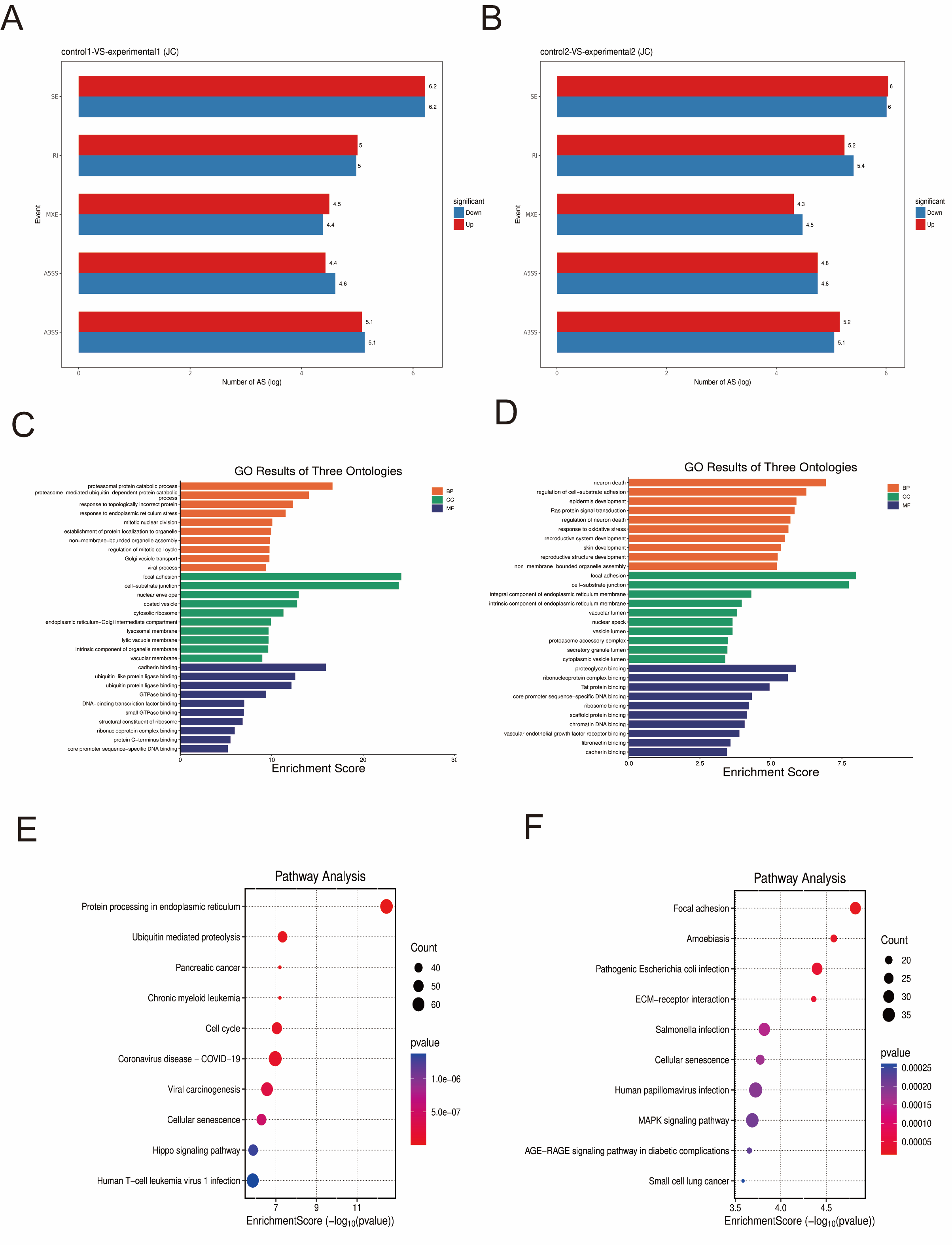

Supplement: Supplementary file 9 [file Image2.tif]

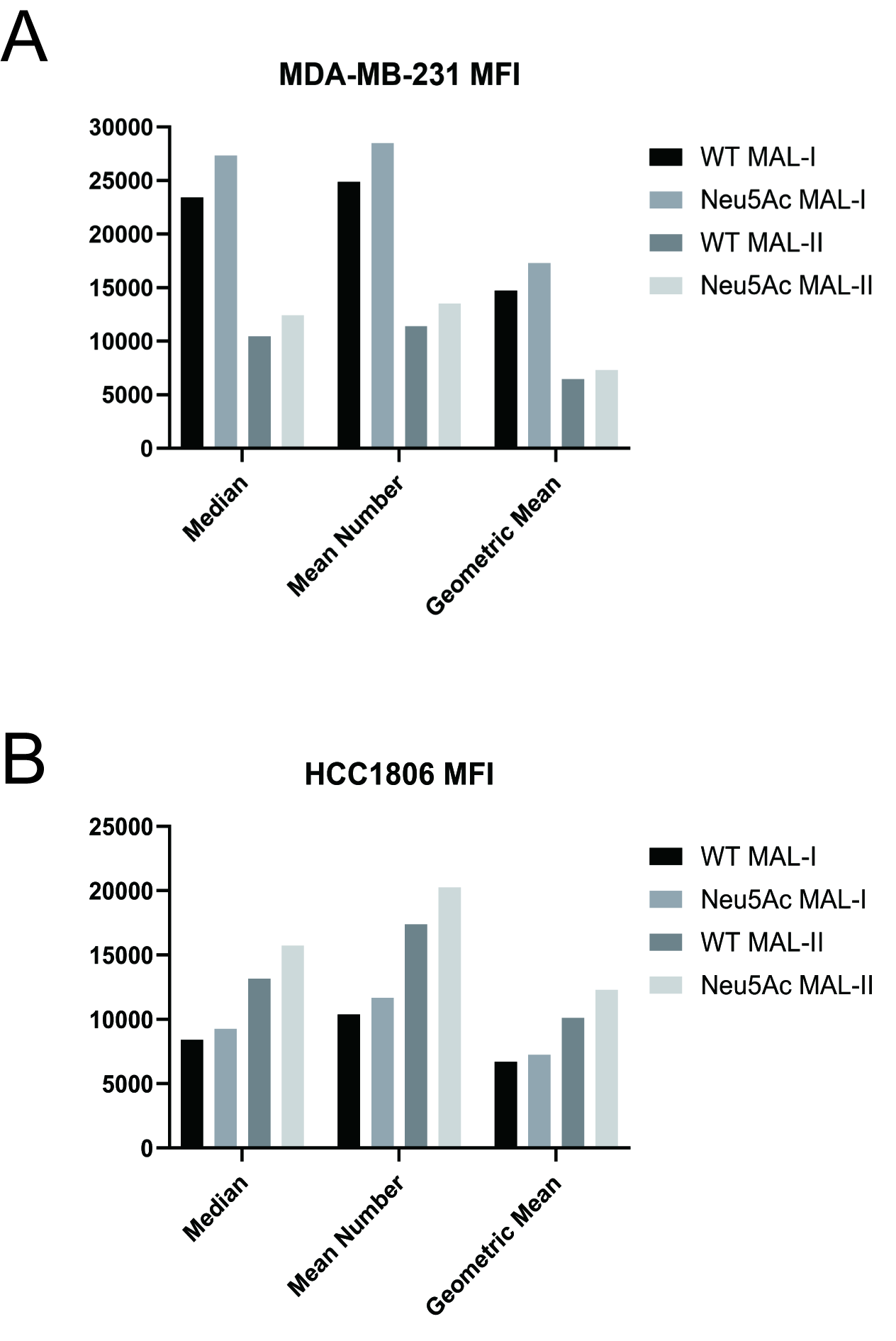

Supplement: Supplementary file 10 [file Image3.tif]
